# Evaluation of the Defect Cluster Content in Singly and Doubly Doped Ceria through In Situ High-Pressure X-ray Diffraction

**DOI:** 10.1021/acs.inorgchem.1c00433

**Published:** 2021-04-30

**Authors:** Cristina Artini, Sara Massardo, Maria Maddalena Carnasciali, Boby Joseph, Marcella Pani

**Affiliations:** † DCCI, Department of Chemistry and Industrial Chemistry, University of Genova, Via Dodecaneso 31, 16146 Genova, Italy; ‡ Institute of Condensed Matter Chemistry and Technologies for Energy, National Research Council, CNR-ICMATE, Via De Marini 6, 16149 Genova, Italy; § INSTM, Genova Research Unit, Via Dodecaneso 31, 16146 Genova, Italy; ∥ Elettra-Sincrotrone Trieste S.C.p.A., ss 14, km 163.5, Basovizza, 34149 Trieste, Italy; ⊥ CNR-SPIN Genova, Corso Perrone 24, 16152 Genova, Italy

## Abstract

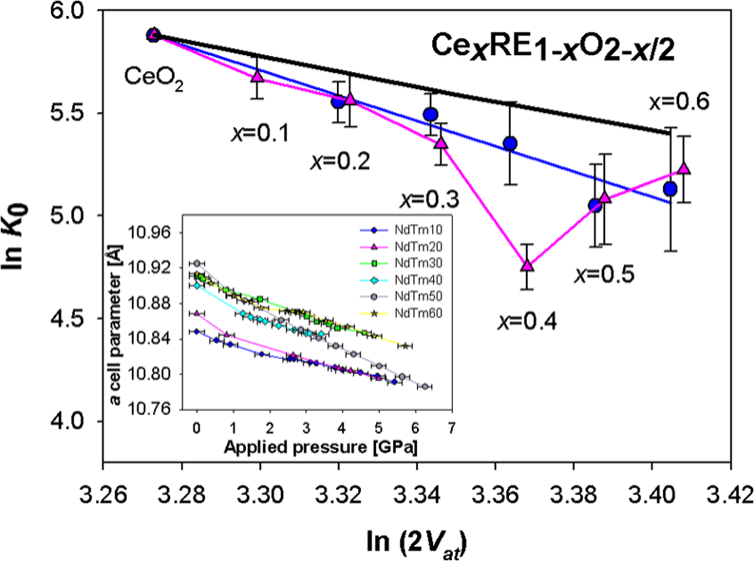

Defect aggregates in doped ceria play a crucial role in blocking the movement of oxygen vacancies and hence in reducing ionic conductivity. Nevertheless, evaluation of their amount and the correlation between domain size and transport properties is still an open issue. Data derived from a high-pressure X-ray diffraction investigation performed on the Ce_1–*x*_(Nd_0.74_Tm_0.26_)_*x*_O_2–*x*/2_ system are employed to develop a novel approach aimed at evaluating the defect aggregate content; the results are critically discussed in comparison to the ones previously obtained from Sm- and Lu-doped ceria. Defect clusters are present even at the lowest considered *x* value, and their content increases with increasing *x* and decreasing rare earth ion (RE^3+^) size; their amount, distribution, and spatial correlation can be interpreted as a complex interplay between the defects’ binding energy, nucleation rate, and growth rate. The synoptic analysis of data derived from all of the considered systems also suggests that the detection limit of the defects by X-ray diffraction is correlated to the defect size rather than to their amount, and that the vacancies’ flow through the lattice is hindered by defects irrespective of their size and association degree.

## Abstract 2

A novel approach to the treatment of high-pressure X-ray diffraction data is applied to several rare earth-doped ceria systems with the aim of providing an evaluation of the amount of defect aggregates and of the composition of the CeO_2_-based solid solution. By this method, it is possible to effectively correlate the structural properties and ionic conductivity of the studied material.

## 1 Introduction

Trivalent rare earth (RE)-doped ceria systems Ce_1–*x*_RE_*x*_O_2–*x*/2_ are currently widely studied as electrolytes in solid oxide fuel and electrolysis cells operating at intermediate temperatures (IT-SOFC and SOEC, respectively) due to their high values of ionic conductivity in the 773–973 K temperature range^1^ and their good chemical compatibility with LSCF, namely the (La,Sr,Co) ferrite diffusely considered as the most efficient material for SOC air electrodes.^2^ Their effectiveness is reported to be driven by numerous strictly interconnected issues, such as the RE^3+^ size and the compositional extent of the CeO_2_-based solid solution,^3,4^ as well as extrinsic factors like the synthetic procedure and the sintering process, both of which influence the microstructural features of the sample.^5^ Nevertheless, the first and foremost cause of hindrance to the movement of oxygen ions through the lattice has to be sought in the occurrence of defect aggregates, which strongly affect the entrance of randomly dispersed RE^3+^ ions into the CeO_2_-based solid solution and ultimately impair the oxide transport properties by blocking the movement of oxygen vacancies through the lattice.

The nature of defects arising in doped ceria is strictly connected to the crystallographic properties of the material. CeO_2_ is characterized by a fluorite-like cubic cell named F belonging to the *Fm*3̅*m* space group^6^ and having four formula units per cell; Ce and O occupy, respectively, the 4*a* (0, 0, 0) and 8*c* (1/4, 1/4, 1/4) atomic sites, and Ce is eight-coordinated to O. The Ce^4+^ partial substitution by a RE^3+^ ion primarily induces the formation of a CeO_2_-based solid solution with isolated RE_Ce_^′^ defects and oxygen vacancies acting as guests; at a sufficiently high temperature, the latter are free to move through the lattice, being thus responsible for ionic conduction. The stability of such a solid solution is guaranteed up to a certain RE^3+^ content, which depends on the RE element, being driven both by the Ce^4+^/RE^3+^ size closeness and by the binding energy of the RE^3+^-vacancy clusters.^3,7^ Beyond the threshold, superstructure peaks referable to the *Ia*3̅ space group appear in the diffraction patterns: they mark the presence of the aforementioned RE^3+^-vacancy clusters, crystallizing in the *Ia*3̅ cubic phase named C, typical of sesquioxides of the heaviest rare earths (Gd-Lu),^8^ where the RE doping ion is six-coordinated to O. If the Ce^4+^/RE^3+^ ionic size mismatch is not too large (namely from RE ≡ Sm to Tm,^9^ as well as in doubly doped systems, where, for instance, RE ≡ (Nd,Dy)^10^ or (Nd,Tm)^11^), a F/C hybrid phase named H occurs, where the F-based solid solution mainly hosts randomly dispersed C clusters.^12^ In the H phase, C microdomains are so intimately interlaced with the F matrix that diffraction peaks common to the F and C phases are perfectly superimposed, and the C cell size is exactly doubled with respect to that of F.^13,14^ If, on the contrary, the Ce^4+^/RE^3+^ size mismatch is too large, a (F + C) two-phase field appears, such as in Lu-doped ceria. For the sake of completeness, the F, C, and H structural models are reported in the Supporting Information. Nevertheless, in spite of the substantial accuracy of this description, the scenario is not simple, since it is well known that a certain number of C-based nano- or microdomains are stable within the F matrix even within the F stability region: local probes such as Raman spectroscopy, for instance, are able to reveal the presence of C clusters at doping amounts much lower than needed by X-ray diffraction.^12^

The building units constituting C defect clusters have been long investigated,^15−18^ resulting to be mainly 1V_O_^••^RE_Ce_^′^ positively charged dimers and 1V_O_^••^2RE_Ce_^′^ neutral trimers,^15^ with a significant predominance of the latter below a threshold temperature placeable at ∼750 K; at higher temperatures, the trimers tend to dissociate due to their lower configurational entropy with respect to the dimers.^19^ The stability of C defect associates is connected to the maximization of the cluster binding energy, which in turn is ruled by the RE^3+^ size and local position with respect to the oxygen vacancy:^7^ according to computational simulations, the binding energy of 1V_O_^••^2RE_Ce_^′^ trimers grows with decreasing RE^3+^ radius if the dopant is located in the nearest-neighbor (NN) position with respect to the vacancy, while it is slightly reduced if it is in the next-nearest-neighbour (NNN) one.^7^ The described evidence thus justifies the minimum in binding energy,^7,15^ as well as the maximum in ionic conductivity, found for RE ≡ Sm^3+^ and Gd^3+^.^4,20^

The crystallographic nature and the spatial extent of the C-based local ordering have been the subject of numerous studies performed by both experimental and theoretical approaches. To this purpose, techniques such as selected area electron diffraction (SAED),^21^ transmission electron microscopy (TEM),^22^ extended X-ray absorption fine structure (EXAFS),^23−25^ Raman spectroscopy,^26,27^ and X-ray total scattering treated by pair distribution function (PDF)^28,29^ were employed, as well as computational simulations.^15,18^ Nevertheless, the actual amount of C aggregates, and consequently the real RE^3+^ content within the F matrix, could hardly be revealed. While in fact it is relatively easy to recognize the position of the F/H or the F/(F + C) boundary through the occurrence of C superstructure peaks in diffraction patterns, and hence the nominal formulation of the oxide marking the F compositional boundary, it is nontrivial to determine how RE^3+^ ions are actually distributed over C and F, i.e., over 1V_O_^••^2RE_Ce_^′^ and 1V_O_^••^RE_Ce_^′^ C domains on one hand and randomly placed RE_Ce_^′^ on the other. Put differently, the actual RE^3+^ maximum content in F does not correspond to the one based on the overall oxide stoichiometry, since a certain amount of RE^3+^ ions are blocked within the C domains, even if this evidence cannot be revealed by X-ray diffraction in a straightforward way. This issue is of special interest, since the compositional extent of the F solid solution based on the nominal composition of the oxide is different for each lanthanide doping ion, with a minimum for RE ≡ Gd and Sm,^3^ and it is often taken as an indicator of the transport properties of the material.

Co-doping of ceria has been accomplished for many systems, such as Gd-Y,^30^ Gd/Sm-,^31^ La/Sm-,^32^ Sm/Nd-,^33^ Nd/Gd-,^34^ La/Dy-,^35^ Gd/Sm/La,^36^ and even others, due to the generally observed lowering of activation energy to ionic conduction^11^ and enhancement of ionic conductivity^30,37,38^ with respect to singly doped ceria. Within this framework, the present research group recently undertook an experimental study of the structural, Raman, and transport properties of two co-doped systems having the same average doping ion size as Sm-doped ceria, namely Ce_1–*x*_(Nd_0.63_Dy_0.37_)_*x*_O_2–*x*/2_^10^ and Ce_1–*x*_(Nd_0.74_Tm_0.26_)_*x*_O_2–*x*/2_.^11^^11^ Sm-doped ceria was chosen as a reference material due to its remarkable ionic conductivity values among ceria-based systems. In comparison to Ce_1–*x*_Sm_*x*_O_2–*x*/2_, crystallographic results derived from the studied co-doped systems point at a widening of the compositional extent of the F stability region, as well as at the occurrence of larger cell parameters. Both of these evidences are believed to be caused by the preferential entrance of the larger RE^3+^, i.e., Nd^3+^, into F, and the smaller RE^3+^, i.e., Dy^3+^ or Tm^3+^, into C defects, in accordance with the aforementioned higher binding energy of C clusters formed by smaller rare earth ions.^7^ Since 1V_O_^••^2RE_Ce_^′^ trimers are known to be responsible, together with 1V_O_^••^RE_Ce_^′^ dimers, for blocking the transport of oxygen vacancies through the lattice, the enlargement of the F region makes in principle co-doped systems very promising in terms of ionic conduction. Indeed, in (Nd,Tm)-doped ceria, two different activation energies to ionic conduction were revealed within different temperature ranges, namely below and above ∼750 K, and very interestingly, the high temperature activation energy resulted to be lower in (Nd,Tm)- than in Sm-doped ceria, as a direct consequence of the distribution of Nd^3+^ and Tm^3+^ ions between the F matrix and C defect aggregates.^11^ Nevertheless, the actual composition of the F solid solution and the amount of C domains are not known.

In this work, a novel approach based on in situ high-pressure synchrotron X-ray diffraction is proposed and applied to Ce_1–*x*_(Nd_0.74_Tm_0.26_)_*x*_O_2–*x*/2_ to provide a reliable evaluation of the amount of C defect aggregates, and hence of the composition of the F phase. In order to have useful terms of comparison, the technique is also applied to high-pressure data of Sm-^39^ and Lu-doped ceria.^40^ The methodological basis of the approach, as thoroughly described in the Discussion section, is founded on the exhaustive work by Anderson and Nafe,^41^ who analyzed a huge amount of oxides and found a linear trend of ln *K*_0_ vs ln(2*V*_at_), with *K*_0_ and *V*_at_ being the zero applied pressure bulk modulus and the mean atomic volume, respectively. By comparing the experimental and expected trends, it is possible to recognize the effect of oxygen vacancies on *V*_at_, to consequently deduce the occupancy factor of the O and RE crystallographic sites, and finally to calculate the amount of C defect aggregates and the RE^3+^ amount actually entering the F structure. The results point at the existence of a strict correlation between the binding energy, amount, and size of C defects. The obtained outcome can help in drawing relevant conclusions regarding the ionic conduction properties of the studied material.

## 2 Experimental Section

### 2.1 Synthesis

Six samples belonging to the Ce_1–*x*_(Nd_0.74_Tm_0.26_)_*x*_O_2–*x*/2_ system (nominal *x* = 0.1, 0.2, 0.3, 0.4, 0.5, and 0.6) were synthesized by oxalate coprecipitation, as described in ref (42); the Nd/Tm ratio was selected in order to reproduce the ionic size of Sm^3+^ with CN 8. Stoichiometric due amounts of Ce (Johnson Matthey ALPHA 99.99 wt %), Nd_2_O_3_ (Alfa Aesar, 99.99 wt %), and Tm_2_O_3_ (Mateck, 99.99 wt %) were separately dissolved in HCl (13 vol %), and the three solutions were mixed. Then, an oxalic acid solution in large excess was poured into the mixtures, causing the immediate precipitation of the mixed Ce/Nd/Tm oxalates, which were then filtered, washed, dried for 12 h, and treated in air at 1373 K for four days to obtain the corresponding mixed oxides with a high crystallinity degree.

### 2.2 Scanning Electron Microscopy–Energy-Dispersive System (SEM–EDS)

Scanning electron microscopy was used to determine the overall lanthanide content of the samples. An electron microscope with a field-emission gun and energy-dispersive system (FE-SEM-EDS, Zeiss SUPRA 40 VP-30–51 scanning electron microscope, equipped with a high-sensitivity “InLens” secondary electron detector and an EDS microanalysis INCA Suite Version 4.09, Oxford Instruments) was employed for this purpose. Samples were pressed, graphite-coated, and observed at a working distance of 15 mm, with an acceleration voltage of 20 kV. EDS analyses were carried out on at least 5 points for each formulation.

### 2.3 High-Pressure Synchrotron X-ray Powder Diffraction (HP-XRPD)

X-ray diffraction patterns were collected at ambient and high pressure at the XPRESS diffraction beamline of the Elettra Synchrotron radiation facility located in Trieste (Italy).^43^ Data were acquired by a monochromatic circular beam with a wavelength of 0.4957 Å and diameter around 50 μm at pressures ranging between 0 and ∼6 GPa by means of a gear-driven Boehler–Almax plate diamond anvil cell (plate DAC) with a large X-ray aperture containing diamonds of culet size 300 μm. Several 200 μm-thick rhenium gaskets were pre-indented using the plate DAC, which allowed to reduce their thickness below 110 μm and to drill a through hole of diameter 100 μm by spark erosion. Specimens were placed into the chamber, and the applied pressure was calibrated by adding Cu and considering the position of the (111) diffraction peak. Silicon oil was used as the pressure-transmitting medium (PTM), as it was able to provide hydrostatic pressure conditions up to the maximum applied pressure. The experimental setup is provided with a MAR345 image plate detector, and images of the diffraction rings were converted into intensity vs 2ϑ plots through the fit2D software.^44^ Data were collected in the 5–25° angular range at least three times after reaching every new value of applied pressure in order to check their reproducibility. The samples were named NdTm10_2.68, NdTm20_4.23, and so on, in accordance with the nominal (Nd,Tm) atomic percent with respect to the total rare earth content, with the applied pressure in GPa.

**Figure 1:**
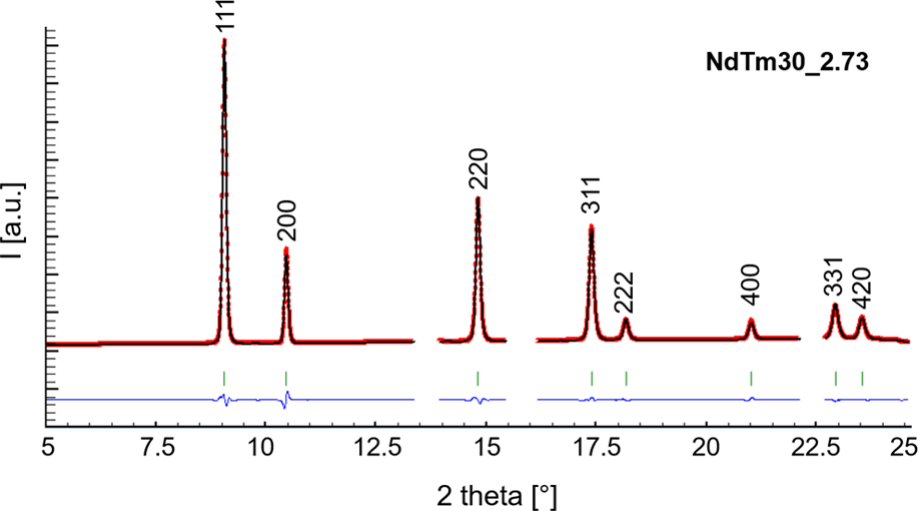
Rietveld refinement plot of sample NdTm30_2.73. The red dotted and the black continuous lines depict the experimental and calculated pattern, respectively; the blue lower line is the difference curve; vertical bars indicate the calculated positions of Bragg peaks. Miller indexes of the F structure (space group *Fm*3̅*m*) are reported. Excluded regions are placed corresponding to the Bragg peaks of Cu.

Structural models were refined through the Rietveld method by the FullProf software.^45^ With specific regard to the refinement procedure, the peak profile was described by a pseudo-Voigt function, the background was refined by interpolating a set of ∼60 points taken from the pattern, and an overall displacement parameter (*B*_ov_) was optimized too. The Ce/Nd/Tm ratio was set at the values provided by EDS and kept fixed, due to the closeness of the atomic scattering factors of the three cited elements. In the last refinement cycle, the lattice parameters, the refinable atomic coordinates, *B*_ov_, the scale factor, five peak parameters, two asymmetry parameters, and the background points were refined. The angular regions where Cu diffraction peaks occur were excluded from the refinements. In Figure 1, the Rietveld refinement plot of sample NdTm30_2.73 is reported as a representative example of the results of structural modeling.

The trend of the lattice volume vs pressure was modeled through the EosFit7-Gui software^46^ by the third-order Vinet EoS,^47^ which is expressed by the following equation
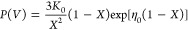
1with
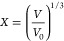
2
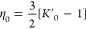
3with
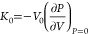
4being the bulk modulus at zero applied pressure, *K*_0_^′^ its first derivative with respect to pressure, and *V*_0_ the cell volume at ambient conditions. The third-order Vinet EoS fits the model to the data by refining *V*_0_, *K*_0_, and *K*_0_^′^; hence, once they reach convergence by least-squares minimization of the difference between experimental and calculated data, the software provides the values of the refined parameters together with their standard deviations.

High-pressure structural results obtained from the present system are compared to the ones derived from Sm-^39^ and Lu-^40^ doped ceria, as well as to those obtained at ambient pressure from the same (Nd,Tm)-doped system.^11^

## 3 Results

EDS analyses performed on the (Nd,Tm)-doped system provided the results reported in Table 1, showing a good agreement between nominal and experimental *x* values. Similar results were found for Sm-^39^ and Lu-^40^ doped ceria.

**Table 1:** Experimental *x* Values of Ce_1–*x*_(Nd_0.74_Tm_0.26_)_*x*_O_2–*x*/2_ Samples

sample	experimental *x*
NdTm10	0.09(1)
NdTm20	0.18(4)
NdTm30	0.26(4)
NdTm40	0.37(5)
NdTm50	0.45(4)
NdTm60	0.57(9)

In full agreement with the outcome of the ambient-pressure X-ray acquisitions,^11^ even at high pressure the F/H crossover is located at *x* slightly lower than 0.6, with the composition Ce_0.4_(Nd_0.74_Tm_0.26_)_0.6_O_1.7_ being the only one displaying C-related peaks. This behavior, also common to the Ce_1–*x*_(Nd_0.63_Dy_0.37_)_*x*_O_2–*x*/2_ system,^10^ marks a substantial difference between these doubly doped systems and Sm-doped ceria, where peaks of the superstructure become visible at *x* ∼ 0.3.^26^

**Figure 2:**
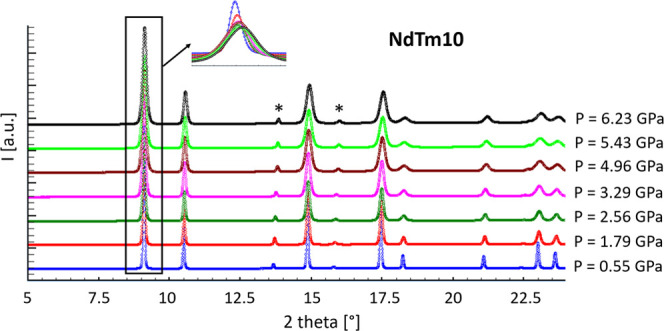
Stacked diffraction patterns of sample NdTm10; asterisks mark the presence of Cu used for pressure calibration. Inset: enlarged view of the main peak.

**Figure 3:**
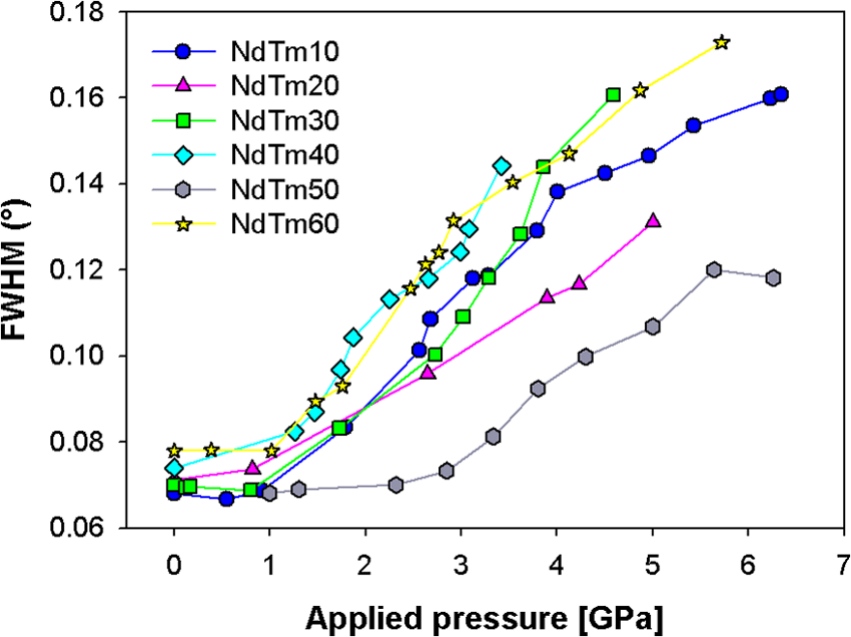
FWHM trend of the most intense reflection, namely (111) for the F model and (222) for the H model, vs applied pressure.

Figure 2 reports the stacked diffraction patterns of sample NdTm10 at different applied pressures, and the inset shows an enlarged view of the main peak as a representative example of the behavior of all of the considered compositions. As already observed in Sm-^39^ and Lu-^40^ doped ceria, no structural changes occur with increasing pressure up to ∼7 GPa. The most important effects exerted on the Bragg peaks by the pressure application are the shift toward higher 2θ values, as well as the decrease of the broadening and intensity. The latter, in particular, can be inferred from the inset to Figure 2, but even more clearly from Figure 3, representing the full-width half-maximum (FWHM) trend of the most intense peak as a function of the applied pressure: a significant FWHM increase occurs starting from ∼1 GPa. The described evidences are related to two effects, namely the reduction of the mean lattice parameter due to compression, which causes the peak shift, and the local distribution of cell sizes, responsible for the peak broadening. Refined lattice parameters and Rietveld agreement factors for each composition at each pressure, as well as crystallographic data of both F and H phases, are reported in the Supporting Information.

**Figure 4:**
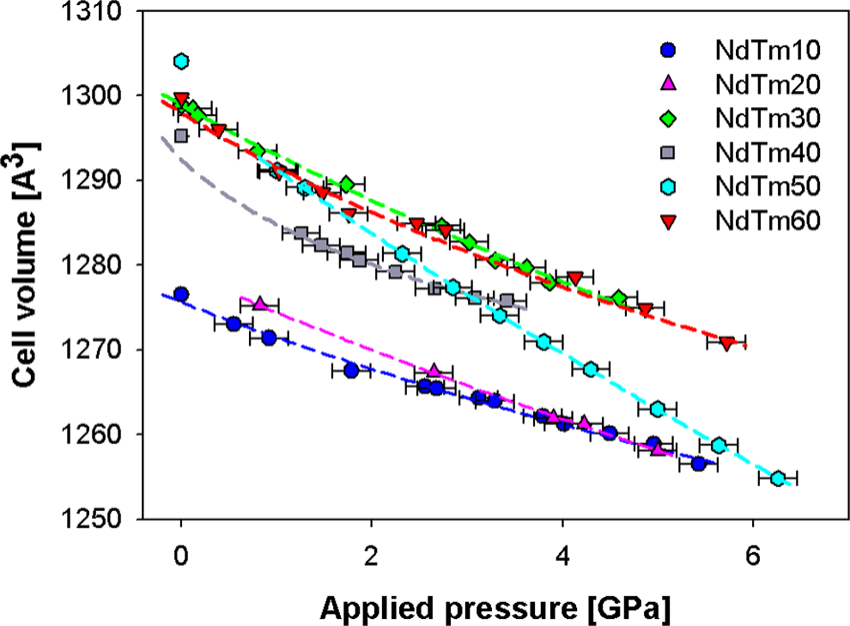
Refined cell volumes as a function of the applied pressure; the third-order Vinet EoS fits the model to the data. Cell volumes of samples crystallizing in the F phase are multiplied by 8 in order to make them comparable to the one assuming the C structure. Vertical error bars are hidden by data markers.

As aforementioned, the trend of the cell volume vs applied pressure was analyzed using the third-order Vinet EoS in order to estimate the bulk modulus at zero pressure (*K*_0_). Graphical results of the fit, appearing in Figure 4, suggest that the cell volume decrease is not linear within the pressure range considered, generally becoming progressively less steep, thus indicating a compressibility reduction with increase in the applied pressure.

**Figure 5:**
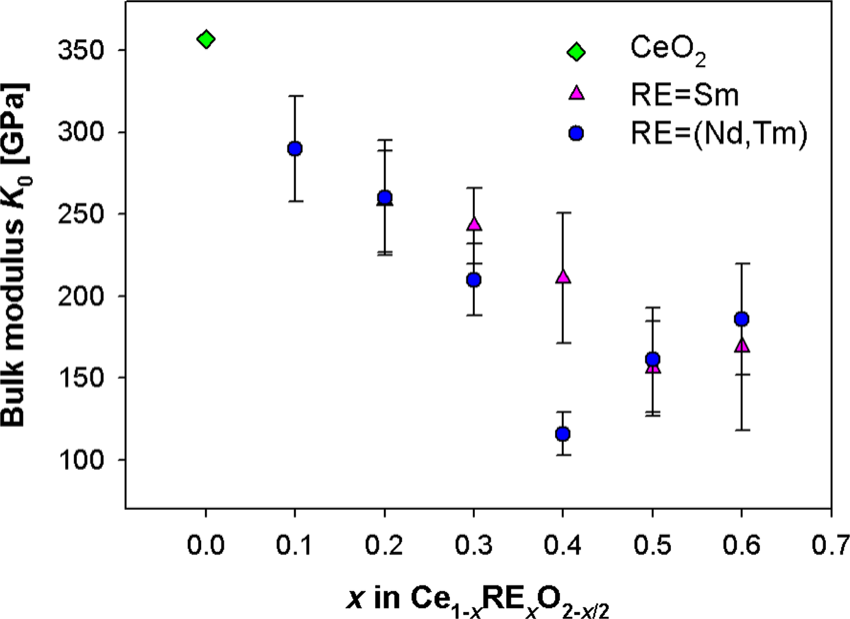
Zero pressure bulk modulus (*K*_0_) vs the doping content for (Nd,Tm)- and Sm-^39^ doped ceria; the CeO_2_ data are taken from ref (48).

The calculated values of *K*_0_ for (Nd,Tm)- and Sm-^39^ doped ceria, as well of CeO_2,_^48^ are reported vs the doping content in Figure 5. A roughly linear decrease can be observed in both systems, together with a slight upturn at the highest *x* values. For the sake of completeness, the *K*_0_^′^ values obtained from the fit are reported in the Supporting Information in Table S3.

## 4 Discussion

Anderson and Nafe analyzed the *K*_0_ values and structural data of a huge variety of ionic crystals, covalent crystals, and oxides,^41^ brilliantly finding the following empirical correlations, valid for ionic crystals (eq 5) and for covalent crystals and oxides (eq 6)
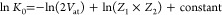
5
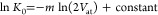
6where *Z*_1_ and *Z*_2_ are the cationic and anionic charge, respectively, in ionic crystals, and 2*V*_at_ the lattice volume divided by the halved number of atoms therein contained; for oxides, *m* occurring in eq 6 ranges between 3 and 4, while for covalent crystals it assumes the value 4/3. In all of the classes of materials, a decreasing linear trend is thus observed, accounting for the progressively increasing compressibility of a solid with increase in the mean atomic volume. Moreover, based on eqs 5 and 6, the oxides result to be characterized by a much stronger dependence of *K*_0_ on the atomic volume than both ionic and covalent crystals.

**Figure 6:**
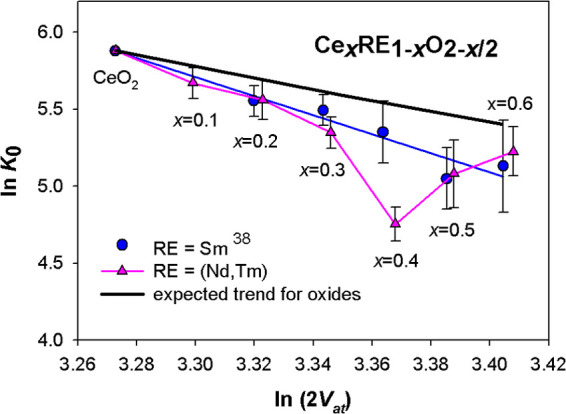
Behavior of ln *K*_0_ vs ln(2*V*_at_). *K*_0_ and *V*_at_ values are derived from EoS fits and Rietveld refinements, respectively. Data of CeO_2_ are taken from ref (48). Cell volumes of samples crystallizing in the F phase are multiplied by 8 in order to make them comparable to those assuming the C structure.

In order to fit the experimental data to the described expected trend, for each composition of (Nd,Tm)-doped ceria the atomic volume *V*_at_ calculated from the oxide stoichiometry was associated to the *K*_0_ value obtained from the EoS: the elaboration provided the results appearing in Figure 6, where also the data of Sm-doped ceria^39^ are reported. The black thick line represents the expected trend for oxides according to Anderson and Nafe,^41^ obtained by attributing to *m* the value 3.5, which is intermediate between the suggested end values,^41^ and constraining the line to pass through the point corresponding to ln *K*_0_ and ln(2*V*_at_) of CeO_2_.^48^ The expected trend is described by the following equation
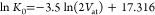
7

At this stage, it is worth mentioning that a possible inaccuracy of the slope value, caused by the absence of hints orienting a more precise choice of *m*, can be at the root of the corresponding slight inaccuracies in the absolute values of the F/C distribution of the RE^3+^ ions, but not in the recognition of the general trend. The data of both systems deviate from the predicted trend toward lower ln *K*_0_ values, showing a much more pronounced dependence of ln *K*_0_ on ln(2*V*_at_), which results in a low doping content at *m* ∼ 6; in addition, while the data of Sm-doped ceria display a roughly linear trend along the whole compositional range, the ones of the doubly doped system present a substantial superposition with the former system up to *x* = 0.3, a significant drop at higher *x*, and a reconnection close to *x* = 0.6.

As aforementioned, the F phase can be defined as a CeO_2_-based solid solution hosting two different guests: the prevailing one consists of isolated RE_Ce_^′^ defects and randomly distributed oxygen vacancies, both of which are accommodated at the F crystallographic positions, namely at the Ce and the O site, respectively; the minor one is constituted of a certain amount of associated RE^3+^ ions and vacancies, mainly giving rise to either 1V_O_^••^RE_Ce_^′^ or 1V_O_^••^2RE_Ce_^′^ C-based clusters. Below the *x* value corresponding to the F limit, the amount of isolated RE_Ce_^′^ defects largely prevails over that of the C defect aggregates; nonetheless, even in the absence of C diffraction peaks, the latter are present, as testified by the occurrence of the typical C band in Raman spectra,^12,27^ as well as by the drop in ionic conductivity in Gd- and Sm-doped ceria at *x* > 0.15–0.20,^49^ namely well below the F boundary as revealed by X-ray diffraction. Similarly, it is reasonable to suppose that even beyond the F limit, both isolated RE_Ce_^′^ defect and C defect aggregates are present, with the latter prevailing over the former.

Since the bulk modulus *K*_0_ derives from the Rietveld refinement of the F cell volumes, it refers to the F phase; thus, the substantial deviation of ln *K*_0_ from the trend typical of oxides suggests that the actual atomic volume does not correspond to the one deduced from the oxide stoichiometry, which is to say that the oxide overall stoichiometry and the actual composition of the F phase do not coincide. Starting from this discrepancy, the proper manipulation of *K*_0_ data provides a hint for the estimate of the C defect amount, and hence of the actual composition of the F phase, as described hereinafter. The observed evidence suggests that in both systems, for each ln *K*_0_ value the F mean atomic volume *V*_at_ is underestimated (i.e., the total number of atoms in F is overestimated) if compared to the expected value as derived from the theoretical trend represented in Figure 6. Therefore, the expected ln 2*V*_at_ value (and consequently the expected *V*_at_) was determined for each composition by inserting the corresponding ln *K*_0_ value into eq 7, which describes the ideal behavior of the oxides. Afterward, the corrected number of atoms per F cell was calculated by dividing the refined zero pressure cell volume (*V*_0_) by the so-obtained *V*_at_. The difference between the stoichiometric and corrected number of atoms per cell represents the amount of RE and O atoms not taking part in the F phase, thus forming the C defect clusters. According to the RE_2_O_3_ stoichiometry of C oxides, out of the total number of atoms forming the C defect clusters, 40% were attributed to RE and 60% to O. In doubly doped ceria, the preferential entrance into C defect clusters of Tm^3+^ with respect to Nd^3+^ was hypothesized as being due to the higher binding energy of C defect aggregates containing smaller RE ions, as discussed in ref (11). An example of this approach is reported in detail in the Supporting Information. Following from the described calculations, Table 2 shows how atoms belonging to a Ce_1–*x*_(Nd_0.74_Tm_0.26_)_*x*_O_2–*x*/2_ formula unit distribute over the F and C phases. For comparison, in Table 3, the distribution of doping ions over F and C in Sm- and Lu-doped ceria is reported too, as resulting from the identical calculations performed on the *K*_0_ data derived from a previous study.^39^ Data are reported up to *x* = 0.60 and 0.40 for Sm- and Lu-doped ceria, respectively; it has to be noticed that in terms of X-ray diffraction, the F region extends up to *x* = 0.30 for the former system and up to *x* = 0.40 for the latter.

**Table 2:** Distribution of Atoms of a Ce_1–*x*_(Nd_0.74_Tm_0.26_)_*x*_O_2–*x*/2_ Formula Unit between the F and the C Phase

sample name	overall nominal oxide composition	composition of the F phase	composition of the C phase
NdTm10	Ce_0.90_(Nd_0.74_Tm_0.26_)_0.10_O_1.95_	Ce_0.90_Nd_0.06_O_1.89_	Nd_0.014_Tm_0.026_O_0.06_
NdTm20	Ce_0.80_(Nd_0.74_Tm_0.26_)_0.20_O_1.90_	Ce_0.80_Nd_0.15_O_1.825_	Tm_0.05_O_0.075_
NdTm30	Ce_0.70_(Nd_0.74_Tm_0.26_)_0.30_O_1.85_	Ce_0.70_Nd_0.22_O_1.73_	Tm_0.08_O_0.12_
NdTm40	Ce_0.60_(Nd_0.74_Tm_0.26_)_0.40_O_1.80_	Ce_0.60_Nd_0.17_O_1.45_	Nd_0.13_Tm_0.10_O_0.35_
NdTm50	Ce_0.50_(Nd_0.74_Tm_0.26_)_0.50_O_1.75_	Ce_0.50_Nd_0.37_Tm_0.02_O_1.58_	Tm_0.11_O_0.17_
NdTm60	Ce_0.40_(Nd_0.74_Tm_0.26_)_0.60_O_1.70_	Ce_0.40_Tm_0.09_Nd_0.26_O_1.32_	Nd_0.18_Tm_0.07_O_0.375_

**Table 3:** Distribution of Atoms of a Ce_1–*x*_Sm_*x*_O_2–*x*/2_ and a Ce_1–*x*_Lu_*x*_O_2–*x*/2_ Formula Unit between the F and the C Phase

sample name	overall nominal oxide composition	composition of the F phase	composition of the C phase
Sm20	Ce_0.80_Sm_0.20_O_1.90_	Ce_0.80_Sm_0.15_O_1.825_	Sm_0.05_O_0.075_
Sm30	Ce_0.70_Sm_0.30_O_1.85_	Ce_0.70_Sm_0.26_O_1.79_	Sm_0.04_O_0.06_
Sm40	Ce_0.60_Sm_0.40_O_1.80_	Ce_0.60_Sm_0.34_O_1.71_	Sm_0.06_O_0.09_
Sm50	Ce_0.50_Sm_0.50_O_1.75_	Ce_0.50_Sm_0.38_O_1.57_	Sm_0.12_O_0.18_
Sm60	Ce_0.40_Sm_0.60_O_1.70_	Ce_0.40_Sm_0.52_O_1.58_	Sm_0.08_O_0.12_
Lu10	Ce_0.90_Lu_0.10_O_1.95_	Ce_0.90_O_1.80_	Lu_0.10_O_0.15_
Lu20	Ce_0.80_Lu_0.20_O_1.90_	Ce_0.80_Lu_0.06_O_1.69_	Lu_0.14_O_0.21_
Lu30	Ce_0.70_ Lu_0.30_O_1.85_	Ce_0.70_Lu_0.15_O_1.625_	Lu_0.15_O_0.225_
Lu40	Ce_0.60_ Lu_0.40_O_1.80_	Ce_0.60_Lu_0.23_O_1.545_	Lu_0.17_O_0.255_

Two general observations can be done at a first glance. First of all, it can be noticed that in each system, even at the minimum considered RE^3+^ amount (*x* = 0.10), a measurable fraction of the doping atoms do not enter the F phase. This fraction becomes larger with increasing *x*, and even beyond the F boundary the composition of the F phase keeps incorporating RE^3+^ ions, thus implying that doping ions spontaneously divide into the F and C phases at each composition. Secondly, in the doubly doped system, the RE^3+^ amount occurring in C is generally higher than in Sm-doped ceria, and the gap between the two systems becomes wider with increasing *x*. In order to get further insight into the latter issue, the data collected from the Lu-doped system^40^ were taken into account too and are reported in the last part of Table 3: at each *x*, an even larger portion of doping ions enters the C domains. This evidence, namely the Lu > (Nd,Tm) > Sm order ruling the tendency toward the formation of C domains, seems thus to follow from the above-mentioned higher binding energy of C defect aggregates made up of smaller rare earth ions, which favors their formation.

A confirmation of the existence of C-based nanodomains even at a very low RE^3+^ amount can be found in the smaller coordination number of RE^3+^ with respect to Ce^4+^ and in the progressive reduction of the RE–O distances with increase in the RE^3+^ amount as revealed by EXAFS^24,50−53^ and total scattering.^28,54^ Moreover, similar conclusions were also reached as a result of the computational simulations performed by the density-functional theory (DFT) method.^55^ Therefore, it is reasonable to hypothesize that even in the studied systems, RE^3+^ ions and oxygen vacancies not entering the F structure tend to aggregate in the C form even at the lowest RE^3+^ concentration considered. In the light of the previous considerations, there is thus reason to wonder why C defect aggregates become detectable by X-ray diffraction when they reach different concentrations, according to the system: in fact, while in Sm-doped ceria at the upper F limit, namely at *x* = 0.30, the composition of the C phase is Sm_0.04_O_0.06_, in (Nd,Tm)-doped ceria, again at the upper F limit, namely at *x* = 0.50, it is Tm_0.11_O_0.17_, and in Lu40 in correspondence of the same limit (*x* = 0.40), it is Lu_0.17_O_0.255_, as inferable from Table 1. In other words, the detectability of C domains is minimum in Lu-doped ceria, and it increases in the order Lu < (Nd,Tm) < Sm. This issue can be overcome by taking into account that the detectability of a diffraction domain is strictly related to the spatial correlation of its structural order: nanodomains of a certain phase occurring within a matrix can be undetectable by X-ray diffraction irrespective of their concentrations provided that they are sufficiently dispersed, namely that their structural order does not exceed the local scale. On this matter, the role of total scattering analyzed by the pair distribution function (PDF) techniqueis essential, since it is able to reveal local distortions invisible to X-ray diffraction. Diffraction domains can be detected when their size goes beyond a certain threshold, i.e., when the domains associate. In this respect, the detectability of C domains and the amount of C-based domains at the upper F limit, which follow opposite orders, suggest that Sm-based C domains are the most prone to associate but the most difficult to form, while the opposite happens for the Lu-based ones, with the (Nd,Tm)-based system showing an intermediate behavior. It can be thus inferred that the driving force for the formation of domains is the defects’ binding energy, while the association of domains favors their stabilization. Considering all these issues, the behavior of C defect aggregates as a function of the RE element seems to resemble that of crystallization nuclei within a liquid mass as a function of temperature. In that case, the critical size of the nuclei decreases and correspondingly the nucleation rate increases, with increasing undercooling; the growth rate, on the contrary, is higher when the undercooling is less pronounced, so that less nuclei form and only the sufficiently large ones are stable. Analogously, in our systems, the higher the binding energy (i.e., the smaller the RE^3+^ ion), the higher the nucleation rate and the smaller the critical size of domains, which explains the occurrence of large amounts of highly dispersed small C domains, not detectable by X-ray diffraction in spite of their numerousness; for lower binding energies (i.e., for larger RE^3+^ ions), the stability of C domains is ensured by their growth beyond a threshold size depending on the RE element, which makes C domains detectable by X-ray diffraction even when they are present in a relatively low amount.

This interpretation is in good agreement with data derived from measurements of the transport properties of the present and similar systems. It is well known that, if extrinsic parameters such as preparation and annealing conditions are neglected, ionic conductivity in doped ceria varies as a function of the chemical nature and concentration of the doping ion. Nonetheless, while the highest conductivity values are strictly correlated to the RE^3+^ nature, being provided by Nd-, Sm-, and Gd-doped ceria,^4^ and in general by proper doubly doped systems,^56^ the dependence on the RE^3+^ amount is roughly the same for each doping ion: ionic conductivity presents in fact a maximum at *x* ranging between 0.10 and 0.25 for Lu-,^57^ (Nd,Tm)-^11^ and Sm-^58^ doped ceria, not differently from Y-doped ceria,^59^ for example. This evidence corroborates the idea that ionic conductivity is negatively affected by the presence of C defect aggregates; their size and hence their detectability by X-ray diffraction, on the contrary, do not play any relevant role. Therefore, it can be concluded that a wide stability region of the F phase, being determined by the high dispersion degree and by the small size of C defect aggregates, is not expected to induce high and exploitable values of ionic conductivity over a correspondingly large compositional extent, as indeed experimentally observed in many doped ceria systems.

## 5 Conclusions

A high-pressure X-ray diffraction study was systematically performed up to ∼6 GPa on six compositions belonging to the Ce_1–*x*_(Nd_0.74_Tm_0.26_)_*x*_O_2–*x*/2_ system (*x* = 0.10–0.60) with the aim of developing a new approach for the evaluation of the defect aggregate content, due to their prominent role in reducing the ionic conductivity of the material. The treatment was extended to data previously collected from Sm- and Lu-doped ceria in order to comparatively discuss the three systems.

Three main conclusions can be drawn from the results of the study.

- Even at the lowest considered *x* value, doping ions form defect aggregates; their amount increases with increasing RE^3+^ content and decreasing RE^3+^ size.
- Small RE^3+^ ions give rise to numerous tiny defect domains, which cannot be revealed by X-ray diffraction due to their reduced spatial correlation until they associate and reach a sufficiently large size. This evidence can be correlated with the defects’ binding energy, which is higher for defects containing smaller doping ions, thus favoring a high nucleation rate in comparison to the growth rate.
- The occurrence of C defect associates even at a very low doping content contributes to clarify the reasons behind the drop in ionic conductivity observed in many Ce_1–*x*_RE_*x*_O_2–*x*/2_ systems starting from *x* = 0.10 to 0.15. The results of this study suggest that the movement of oxygen vacancies through the lattice is hindered by the presence of defects irrespective of their size.

## Supporting Information Available

Structural models of the F, H and C phases; refined cell parameters and agreement factors of Rietveld refinements; *K*_0_^′^ values obtained from the fit of the model to data by the third order Vinet EoS; example of calculation of the distribution of RE^3+^ ions over the F and the C phase; crystallographic data of the F and the H phase (PDF)
