# Supplementary material for: Evaluation of the Defect Cluster Content in Singly and Doubly Doped Ceria through In Situ High-Pressure X-ray Diffraction
Source: Inorg Chem. 2021 Apr 30;60(10):7306–14. doi: 10.1021/acs.inorgchem.1c00433 (PMC8277124; doi:10.1021/acs.inorgchem.1c00433)
Supplement: Supplementary file 1 — ic1c00433_si_001.pdf [file ic1c00433_si_001.pdf]

Supporting Information

Evaluation of the defect clusters content in singly- and doubly-doped ceria through *in-situ* high pressure x-ray diffraction

Cristina Artini [a, b, *], *Sara Massardo* [a], *Maria Maddalena Carnasciali* [a,c],

Boby Joseph [d], *Marcella Pani* [a, e]

[a] DCCI, Department of Chemistry and Industrial Chemistry, University of Genova, Via Dodecaneso 31, 16146 Genova, Italy

[b] Institute of Condensed Matter Chemistry and Technologies for Energy, National Research Council, CNR-ICMATE, Via De Marini 6, 16149 Genova, Italy

[c] INSTM, Genova Research Unit, Via Dodecaneso 31, 16146 Genova, Italy

[d] Elettra - Sincrotrone Trieste S.C.p.A., ss 14, km 163.5, 34149 Basovizza, Trieste, Italy

[e] CNR-SPIN Genova, Corso Perrone 24, 16152 Genova, Italy

* artini@chimica.unige.it

**CeO_2**<br>F structure<br>*cF*12 *Fm*-3*m* Z=4<br>a=5.4097(1) Å			**$Ce_{1-x}(Nd_{0.74}Tm_{0.26})_xO_{2-x/2}$**<br>Hybrid model<br>for Rietveld refinement<br>of sample with *x*= 0.6<br>*cI*96 *Ia*-3 Z=32			**Tm_2O_3**<br>C structure<br>*cI*80 *Ia*-3 Z=16<br>a=10.4908(1) Å		
Atom	Wyckoff site	Coordinates	Atom	Wyckoff site	Coordinates	Atom	Wyckoff site	Coordinates
Ce	4*a*	0, 0, 0	Ce/Nd/Tm1	24*d*	*x*, 0, 1/4<br>*x* = 0.25	Tm1	24*d*	*x*, 0, 1/4<br>*x* = 0.2839
			Ce/Nd/Tm2	8*a*	0, 0, 0	Tm2	8*a*	0, 0, 0
O	8*c*	1/4, 1/4, 1/4	O1	48*e*	*x*, *y*, *z*<br>*x* = 0.125<br>*y* = 0.375<br>*z* = 0.125	O	48*e*	*x*, *y*, *z*<br>*x* = 0.096<br>*y* = 0.361<br>*z* = 0.129
			O2	16*c*	*x*, *x*, *x*<br>*x* = 0.125			

Table S1 - Hybrid structural model compared to the F model typical of CeO_2 and the C model typical of sesquioxides of heavy rare earths, such as Tm_2O_3. Data of CeO_2 are taken from C. Artini *et al.*, *Inorg. Chem.* 2015, **54**, 4126-4137; data of Tm_2O_3 are taken from J.L. Blanusa *et al.*, *J. Magn. Magn. Mater.* 2000, **213**, 75-81.

Sample	P [GPa]	*a* cell parameter [Å]	R_B	χ^2
NdTm10	0	5.42392(3)	0.78	0.25
	0.55	5.41899(3)	1.04	0.24
	0.92	5.41663(3)	1.04	0.25
	1.79	5.41109(9)	1.15	1.16
	2.56	5.4084(2)	1.44	1.72
	2.68	5.4082(2)	1.63	2.64
	3.12	5.4066(3)	3.89	5.21
	3.29	5.4061(2)	1.86	2.78
	3.79	5.4034(3)	1.95	3.16
	4.01	5.4022(3)	2.18	2.82
	4.5	5.4006(3)	2.16	3.38
	4.96	5.3990(3)	2.05	3.21
	5.43	5.3954(4)	2.04	2.82
	6.23	5.3882(4)	1.98	2.75
	6.34	5.3881(4)	2.03	3.24
NdTm20	0	5.43434(4)	0.31	0.49
	0.82	5.42209(4)	0.43	0.96
	2.65	5.41083(7)	0.96	0.81
	3.90	5.4031(1)	1.11	0.96
	4.23	5.4021(1)	1.14	0.94
	5.00	5.3977(2)	1.38	1.22
NdTm30	0	5.45538(3)	0.48	0.32
	0.12	5.45482(3)	0.31	0.39
	0.17	5.45374(3)	0.43	0.41
	0.80	5.44773(3)	1.33	0.70
	1.73	5.44226(5)	0.39	4.28
	2.73	5.43535(9)	0.46	0.41
	3.02	5.4327(1)	0.57	0.51
	3.29	5.4297(1)	0.79	0.58
	3.62	5.4284(2)	0.95	0.60
	3.86	5.4259(2)	1.04	0.56
	4.59	5.4229(3)	0.83	0.47
NdTm40	0	5.45022(5)	0.43	0.65
	1.26	5.43416(5)	0.44	0.25
	1.47	5.43211(6)	0.43	0.23
	1.74	5.43089(8)	0.43	0.35
	1.87	5.4296(1)	0.45	0.39
	2.25	5.4276(1)	0.49	0.39
	2.66	5.4249(2)	0.41	0.28
	2.99	5.4231(2)	0.50	0.27
	3.08	5.4233(2)	0.54	0.32
	3.42	5.4228(2)	0.53	0.34
NdTm50	0	5.46269(5)	0.33	0.21
	1.00	5.4446(1)	0.61	0.05
	1.30	5.4417(1)	0.26	0.04
	2.32	5.4307(1)	0.28	0.02
	2.85	5.4251(1)	0.77	0.02
	3.34	5.4204(1)	0.27	0.05

	3.80	5.4160(1)	0.31	0.04
	4.30	5.4114(1)	0.66	0.06
	5.00	5.4047(1)	0.63	0.05
	5.64	5.3986(1)	0.35	0.05
	6.26	5.3930(1)	0.52	0.05
NdTm60	0	10.91314(7)	1.26	1.31
	0.39	10.90262(7)	1.17	1.10
	1.02	10.88856(7)	1.17	0.72
	1.48	10.8817(1)	0.97	0.43
	1.76	10.8749(1)	1.02	0.40
	2.47	10.8714(2)	0.66	0.41
	2.63	10.8709(2)	0.32	0.39
	2.77	10.8692(2)	0.27	0.37
	2.92	10.8699(3)	0.26	0.31
	3.54	10.8611(3)	0.30	0.46
	4.13	10.8537(3)	0.37	0.38
	4.87	10.8432(4)	0.33	0.33
	5.72	10.8320(5)	0.34	0.26

Table S2 – Refined cell parameters and agreement factors of Rietveld refinements performed at different applied pressures of samples belonging to the $Ce_{1-x}(Nd_{0.74}Tm_{0.26})_xO_{2-x/2}$.

Sample	K'_0
NdTm10	34(14)
NdTm20	18(3)
NdTm30	31(12)
NdTm40	121(17)
NdTm50	7(9)
NdTm60	37(16)
Sm20	-2(2)
Sm30	23(2)
Sm40	68(6)
Sm50	39(2)
Sm60	46(2)
Lu10	24(6)
Lu20	13(2)
Lu30	7(1)
Lu40	5(3)

Table S3 – Values of K'_0 (first derivative of K_0 with respect to pressure) deriving from fitting the model to data by the third order Vinet EoS.

Example of calculation of the distribution of RE^{3+} ions over the F and the C phase

NdTm50

$K_0 = 161$ GPa (from the fit of the refined cell volumes *vs.* applied pressure)

$lnK_0 = 5.081$

$\ln K_0 = -3.5 \ln(2V_{at}) + 17.316$ (equation of the line describing the ideal behaviour of oxides, assuming $m = -3.5$).

For $lnK_0 = 5.081$, $\ln(2V_{at}) = 3.496$ → $V_{at} = 16.485$ Å^3/atom

Nominal formula unit of the composition NdTm50: $Ce_{0.50}Nd_{0.37}Tm_{0.13}O_{1.75}$

Each formula unit contains (0.50 + 0.37 + 0.12 + 1.75) = 2.75 atoms.

Each cell contains 2.75 * 4 = 11 atoms, being $Z = 4$.

$V_0 = 163.013$ Å^3 (refined cell volume at ambient pressure)

$\frac{163.013}{16.485} = 9.485$ atoms/cell

$11 - 9.89 = 1.11$ (number of atoms/cell entering C defects)

Due to the higher binding energy of defects containing smaller rare earths, the preferential entrance of Tm^{3+} into C clusters with respect to Nd^{3+} is hypothesized.

Considering the ratio 2/3 of Tm atoms/O atoms in the sesquioxide Tm_2O_3, out of 1.11 atoms entering C defect clusters for each cell,

$1.11 * \frac{2}{5} = 0.44$ Tm atoms/cell and $1.11 * \frac{3}{5} = 0.67$ O atoms/cell enter C defect clusters.

Considering that $Z = 4$, $\frac{0.44}{4} = 0.11$ Tm atoms/formula unit and $\frac{0.67}{4} = 0.17$ O atoms/formula unit enter C defect clusters.

Therefore, considering one formula unit of the overall oxide, $Tm_{0.11}O_{0.17}$ is the composition of the C phase, and (by subtraction), $Ce_{0.50}Nd_{0.37}Tm_{0.02}O_{1.58}$ is the composition of the F phase.

Crystallographic Data (F phase)

Source synchrotron

Chemical formula $Ce_{1-x}(Nd_{0.74}Tm_{0.26})_xO_{2-x/2}$ (x = 0.1, 0.2, 0.3, 0.4, 0.5)

Formula weight 172.37-173.38 g/formula unit

Temperature room temperature

Pressure 0-6 GPa

Wavelength 0.4957 Å

Crystal system cubic

Space group (No.) $Fm\bar{3}m$ (225)

a, b, c, α, β, γ a = b = c = 5.42-5.46 Å; α = β = γ = 90°

V (A^3) 159.22-162.77 $Å^3$

Z 4

d-space range 1.2-3.1 Å

χ^2 see Table S2

R_p 2.5

R_{wp} 3.3

Crystallographic Data (H phase)

Source synchrotron

Chemical formula $Ce_{1-x}(Nd_{0.74}Tm_{0.26})_xO_{2-x/2}$ (x = 0.6)

Formula weight 167.24 g/formula unit

Temperature room temperature

Pressure 0-6 GPa

Wavelength 0.4957 Å

Crystal system cubic

Space group (No.) $Ia\bar{3}$ (206)

a, b, c, α, β, γ a = b = c = 10.90 Å; α = β = γ = 90°

V (A^3) 1295.03 $Å^3$

Z 32

d-space range 1.1-5.4 Å

χ^2 see Table S2

R_p 5.2

R_{wp} 5.9
